# Prudent Use of Tylosin for Treatment of *Mycoplasma gallisepticum* Based on Its Clinical Breakpoint and Lung Microbiota Shift

**DOI:** 10.3389/fmicb.2021.712473

**Published:** 2021-09-09

**Authors:** Anxiong Huang, Shuge Wang, Jinli Guo, Yufeng Gu, Jun Li, Lingli Huang, Xu Wang, Yanfei Tao, Zhenli Liu, Zonghui Yuan, Haihong Hao

**Affiliations:** ^1^National Reference Laboratory of Veterinary Drug Residues (HZAU) and MAO Key Laboratory for Detection of Veterinary Drug Residues, Wuhan, China; ^2^MOA Laboratory for Risk Assessment of Quality and Safety of Livestock and Poultry Products, Huazhong Agricultural University, Wuhan, China; ^3^National Center for Veterinary Drug Safety Evaluation, College of Veterinary Medicine, China Agricultural University, Beijing, China; ^4^Institute of Food Safety and Nutrition, Jiangsu Academy of Agricultural Sciences, Nanjing, China

**Keywords:** *Mycoplasma gallisepticum*, tylosin, dosage regimen, clinical breakpoint, lung microbiota

## Abstract

The aim of this study was to explore the prudent use of tylosin for the treatment of chronic respiratory infectious diseases in chickens caused by *Mycoplasma gallisepticum* (MG) based on its clinical breakpoint (CBP) and its effect on lung microbiota. The CBP was established based on the wild-type/epidemiological cutoff value (CO_WT_/ECV), pharmacokinetics-pharmacodynamics (PK-PD) cutoff value (CO_PD_), and clinical cutoff value (CO_CL_) of tylosin against MG. The minimum inhibitory concentration (MIC) of tylosin against 111 MG isolates was analyzed and the CO_WT_ was 2 μg/ml. M17 with MIC of 2 μg/ml was selected as a representative strain for the PK-PD study. The CO_PD_ of tylosin against MG was 1 μg/ml. The dosage regimen formulated by the PK-PD study was 3 days administration of tylosin at a dose of 45.88 mg/kg b.w. with a 24-h interval. Five different MIC MGs were selected for clinical trial, and the CO_CL_ of tylosin against MG was 0.5 μg/ml. According to the CLSI decision tree, the CBP of tylosin against MG was set up as 2 μg/ml. The effect of tylosin on lung microbiota of MG-infected chickens was analyzed by 16S rRNA gene sequencing. Significant change of the lung microbiota was observed in the infection group and treatment group based on the principal coordinate analysis and the Venn diagrams of the core and unique OTU. The phyla *Firmicutes* and *Proteobacteria* showed difference after MG infection and treatment. This study established the CBP of tylosin against MG. It also provided scientific data for the prudent use of tylosin based on the evaluation of MG infection and tylosin treatment on the lung microbiota.

## Introduction

*Mycoplasma gallisepticum* (MG) is one of the main pathogenic *mycoplasma* in poultry (Raviv and Kleven, 2009), which can cause reproductive and/or respiratory diseases (Ferguson et al., 2003; Vogl et al., 2008; Wang et al., 2021), and causes huge economic losses to the global poultry industry (Kleven et al., 2000; Nascimento et al., 2005; Ley, 2008; Szczepanek et al., 2010; Pflaum et al., 2018). MG mainly infects turkeys and chickens, but it has also been reported in other birds (Sawicka et al., 2020); it can be transmitted horizontally through direct or indirect contact or vertically through eggs (Kleven, 2008). Although vaccination is effective for MG at present (Bennett et al., 2013; Condello et al., 2020; Arachchige et al., 2021), vaccination against MG is not prevalent due to the variation of bacterial strains and the lack of means to prevent horizontal infection (Feberwee et al., 2006).

Antibacterial chemotherapy is the preferred method to control MG infection (Giguere et al., 2006). Macrolides, quinolones, tetracyclines, and *Pleurotus* truncatus have good antibacterial effects on MG (An et al., 2019). Tylosin has a good therapeutic effect on the respiratory diseases of livestock and poultry (Jordan et al., 1999). Extensive use of antibiotics increases antibiotic resistance and raises public health burdens (Gerchman et al., 2008; Gharaibeh and Al-Rashdan, 2011; Boeckel et al., 2019). In order to minimize and contain development of tylosin resistance, it is necessary to carry out antimicrobial resistance monitoring and establish clinical breakpoint (CBP). Moreover, the effect of tylosin on the lung microbiota of chickens infected with MG is unknown and needs to be evaluated.

Clinical breakpoint is the standard to judge whether bacteria are sensitive or resistant to antimicrobial agents (Toutain et al., 2017), which can guide clinical rational drug use and minimize the emergence of bacterial resistance (Tenover, 2006; Strasfeld and Chou, 2010). The European Veterinary Subcommittee on Antimicrobial Susceptibility Testing (VetCAST) (Kahlmeter and Brown, 2004) and the Clinical and Laboratory Standards Institute (CLSI/VAST) (Clinical And Laboratory Standards Institude, 2013) are establishing CBPs for veterinary antimicrobial agents based on wild-type/epidemiological cutoff value (CO_WT_/ECV), pharmacokinetics-pharmacodynamics (PK-PD) cutoff value (CO_PD_), and clinical cutoff value (CO_CL_) (Mouton, 2002). Therefore, MIC distribution, PK-PD data, antimicrobial resistance mechanism, and the clinical data are needed for establishment of CBPs (Brown et al., 2016). At present, there is no CBP of veterinary antimicrobials for the treatment of MG in the world.

Wild-type/epidemiological cutoff value is used for epidemiological surveillance of drug resistance (Smith et al., 2016). The analysis of bacterial MIC data and determination of the ultimate CO_WT_ are mainly determined by software statistical methods (Kronvall et al., 2006; Turnidge et al., 2010; Canton et al., 2012). CO_PD_ links the metabolic process of drugs in animals with PD data *in vitro*, so as to predict the PD results of different doses of drugs in animals and effectively guide clinical medication (Turnidge and Paterson, 2007; Papich, 2014). The Sigmoid E_max_ model is used to replace the traditional dosage regimen (Xiao et al., 2015); it can simulate the interaction between different systems at the target location, and is an important tool for formulating and optimizing dosage regimen (Nielsen and Friberg, 2013; Isla et al., 2015; Ijaz et al., 2016). The CO_CL_ is obtained by comparing the clinical efficacy of different MIC pathogens (Schwarz et al., 2008). At present, the analysis methods of clinical data are still in the exploratory stage, mainly including “Window” calculation (Turnidge and Martinez, 2017), non-linear regression (Toutain et al., 2002), and binary tree analysis (Zheng et al., 2016).

Many studies have used 16S rRNA gene analysis to confirm the importance of human respiratory tract bacterial community in maintaining respiratory health (Man et al., 2017). However, there are many studies on the flora of digestive tract in chickens (Olsen et al., 2008; Li et al., 2017), and few studies on the flora of respiratory tract. Existing studies have shown that the lung microbiota of chickens is related to the environment and breed, as well as the age and sampling location of chickens (Shabbir et al., 2015; Glendinning et al., 2017; Zhou et al., 2020). However, no studies have shown the effects of the drug on the lung microbiota of chickens. Therefore, it is very important to understand the effect of specific antibiotics on the lung microbiota in order to choose the most effective treatment.

In order to minimize and contain the emergence of antimicrobial resistance and guide the prudent and rational use of tylosin, this study established a CBP of tylosin against MG in referring to CLSI and EUCAST. Additionally, 16S rDNA sequencing was also used to evaluate the effect of tylosin in the treatment of MG infection on chicken lung microbiota.

## Materials and Methods

### Chemicals and Reagents

Tylosin tartrate standard (>94.1%) was purchased from Dr. Ehrenstorfer (Augsburg, Germany); tylosin tartrate raw material powder (≥98%) was purchased from Hebei Jiupeng Pharmaceutical Co., Ltd.; ampicillin (≥98%) was purchased from Beijing solab Technology Co., Ltd., and all the chemical reagents and organic solvents used were HPLC grade.

### Animals and Animal Ethics

The SPF eggs used in the toxicity test were purchased from Beijing Bolinger Hamlet Biotechnology Co., Ltd., Three-week-old ordinary white-feather broilers were adapted for 1 week before the PK study. The use of chickens and all experimental protocols in this study were approved by the Institutional Animal Care and Use Committee at HZAU (HZAUSW-2018-052). All procedures regarding animal care and testing were carried out according to the recommendation for the care and use of laboratory animals of Hubei provincial public service facilities.

### Bacterial Strain Isolation and Establishment of CO_WT_

The 111 MG strains were collected from clinical isolates and deposited in the National Reference Laboratory of Veterinary Drug Residues (HZAU). The isolates were identified by polymerase chain reaction (PCR).

According to the characteristics of acid production by metabolizing glucose, an acid–base indicator was added to the culture medium to observe the change of pH, and the content of MG was determined by sampling the color change unit (CCU). *Staphylococcus aureus* (ATCC 29213) was used as the quality control strain. The minimum inhibitory concentration (MIC) of tylosin against clinical isolation of MG was determined by broth microdilution method.

The results of MICs were analyzed by ECOFFinder software to set up the CO_WT_. The final critical value should include at least 95% of the wild-type strains.

### Selection of Strains and *in vitro* PD Study of M17

All MIC_90_ strains were tested for chicken embryo infection, and sterilized FM-4 medium was used as a negative control group. The number and time of dead embryos were recorded.

Minimum inhibitory concentration and minimum bactericidal concentration (MBC) of tylosin against MG M17 in FM-4 broth were determined by broth microdilution method, mutant prevention concentration (MPC) was determined by agar dilution method, and the post-antimicrobial effect (PAE) was determined by the method of bacterial and drug removal after incubation.

The CCU of M17 in FM-4 broth was measured at different time points. The growth curve was measured three times, and the log_10_ average of bacterial concentration was taken as the longitudinal axis and time was taken as the horizontal axis to draw the growth curve. The growth curve of MG in chicken plasma and lung tissue was similar to that in FM-4 broth.

### PK Animal Experiment Design in Chickens

The PK study involved 3-week-old ordinary white-feather broilers. The chickens in the healthy group were given 25 mg/kg b.w. at a single dose by gavage. The plasma and lung tissues were collected at 0, 0.33, 0.67, 1, 1.5, 2, 3, 4, 6, 8, 12, 24, 36, and 48 h after administration, with four chickens at each time point. The chickens in the infected group were infected with MG M17; 1 ml MG solution was injected into the trachea, and the infection dose was 1 × 10^9^ CCU for 7 days. When compared with the healthy group, chickens in the infected group showed obvious symptoms, such as coughing, sneezing, dull breathing, or discharge of serous nasal fluid. When the PCR reaction process was positive, the plasma and lung tissues were collected and stored at −20°C. The time points and number of chickens were consistent with the healthy group.

### Quantitation Analysis of Tylosin in Plasma and Lung Tissue by HPLC

Plasma sample (0.5 ml) was added to 2 ml of acetonitrile and vortexed for 1 min. The solution was centrifuged at 10,000 rpm at 4°C for 10 min; then, the supernatant was removed, and the extraction was repeated. The supernatants were combined and evaporated to near dryness at 45°C under a gentle stream of nitrogen. The residue was suspended in 0.5 ml of mobile phase. Finally, the mixture was vortexed for 30 s and filtered through a 0.22-μm syringe filter prior to HPLC analysis.

Lung tissue samples (1 g) were homogenized and 4 ml of acetonitrile was added; then, the mixture was vortexed and placed on a shaking table for 10 min, the supernatant was removed, and the extraction was repeated. The 0.5-ml 10% zinc acetate solution was added to the suspension and then centrifuged at 10,000 rpm at 4°C for 10 min. The supernatants were combined and evaporated to near dryness at 45°C under a gentle stream of nitrogen. The residue was suspended in 2 ml of methanol aqueous solution twice. The sample was then applied to an HLB cartridge that had been preconditioned with 3 ml of methanol and 3 ml of water. The sample was loaded and washed with 3 ml of 5% ammonia and 3 ml of water and then dried using a low positive pressure. The column was eluted using 6 ml of acetonitrile. The eluate was evaporated to dryness under a gentle stream of nitrogen at 45°C, and the residue was dissolved in 1 ml of mobile phase and filtered as per above.

Tylosin in plasma and lung tissue was determined by a standard external method and identified by HPLC. SB-Aq liquid phase column (250 mm × 4.6 mm, i.d., 5 micron, Agilent, United States) was used for high-performance liquid chromatography. The detection wavelength was 282 nm, and the temperature was 30°C. The mobile phase consists of 0.1% ammonium formate (phase A) and acetonitrile (phase B). The flow rate of the mobile phase is 1 ml/min, and the elution gradient is 0 min (A70:B30)–8 min (A70:B30)–15 min (A0:B100)–15.1 min (A70:B30)–20 min (A70:B30).

The limit of detection (LOD) and the limit of quantification (LOQ) was 0.018 μg/ml and 0.060 μg/ml in plasma, respectively. The LOD and the LOQ was 0.018 and 0.061 μg/g in lung tissue, respectively. Standard curves were linear from 0.1 to 10 μg/ml in plasma (R^2^ = 0.9998) and linear from 0.1 to 10 μg/g in lung tissue (R^2^ = 0.9998). The recovery of tylosin in plasma and lung tissue ranged from 90.57 to 94.25% and 85.37 to 92.43%, respectively. The inter-day variation for determination in plasma and lung tissue ranged from 0.90 to 2.55% and 1.85 to 2.98%, respectively.

The parameters of PK were calculated by WinNonlin software (version 5.2.1, Pharsight Corporation, Mountain View, CA, United States) from the concentration of tylosin in plasma and lung tissue. In order to select a suitable PK model, drug concentration was recorded in the semi-logarithmic graph. The PK parameters obtained by least square regression analysis were calculated by WinNonlin software.

### PK-PD Integration Analysis

The protein binding rate of tylosin in lung tissue was determined by the equilibrium dialysis method. The formula for protein binding rate was protein binding rate = (Dt–Df)/Dt × 100%. Dt was the drug concentration in dialysis bag and Df was the drug concentration in dialysis fluid.

After animal experiments, PK data of lung tissue were processed by WinNonlin 5.2 software, and the primary absorption two-compartment model was selected to calculate the main PK-PD parameters.

Inhibitory Sigmoid E_max_ model is usually used to predict the relationship between PK-PD parameters and antimicrobial effects *in vivo*, which is the PD target. The equation is: $E=E_{\max}-\frac{\left(E_{\max}-E_{0}\right)\cdot C^{N}}{C^{N}+E\,C_{50}^{N}}$. E is the summary PD endpoint, and E_0_ is the effect representing the value of the PD endpoint without drug treatment (i.e., the value of the summary endpoint when the PK-PD index is 0). C is one of the three PK-PD parameters as defined above, and E_max_ is the maximum effect (in relation to E_0_) indicated by the plateau where increased exposures result in no further kill. EC_50_ is the magnitude of C that is needed to achieve 50% of E_max_, and N is the sigmoidicity factor. The PD target under different efficiency [E = 0, –3, and –4 (bacteriostasis, bactericidal and eradication)] was determined with Sigmoid E_max_ equation.

### Dose Estimations

The formulas for calculating dosage using PK-PD parameter target values are as follows: $D\,o\,s\,e=\frac{C\,L\times {\left(A\,U\,C/M\,I\,C\right)}_{B\,P}\times M\,I\,C}{F\times f\,u}$. CL refers to the clearance rate of tylosin in chicken lung tissue; (AUC/MIC)_BP_ refers to the endpoint of PK-PD parameters corresponding to different therapeutic effects; MIC refers to the MIC of clinical MG; F refers to the bioavailability; and fu: the proportion of free drug concentration.

The Mlxplore software was used to simulate and predict the growth of bacteria at three doses (preventive dose, therapeutic dose, and eradication dose) and at different intervals of administration, so as to obtain the best dosage regimen and interval of administration.

### Establishment of CO_PD_

After determining the population distribution of PK-PD parameters, the population distribution of PK-PD parameters, and the endpoint of PK-PD parameters required to achieve different therapeutic effects, the range and corresponding frequency of parameters needed for dose calculation formula were defined in Crystal Ball software (ICON, United States). The Monte Carlo simulation operation times were set to 10,000 to simulate dose distribution. The maximum MIC of 90% Target Attainment Ratio (TAR) is CO_PD_. The determination of PD objectives is mainly based on the semi-*in vivo* bactericidal curve. Using the PD model and Sigmoid E_max_ equation in WinNonlin software, the PK-PD parameters at E = –3 are calculated, which are the PD objectives.

### Establishment of Fluorescence Quantitative PCR

Establishment of fluorescence quantitative PCR method: Through cloning of recombinant plasmid, restriction enzyme identification, plasmid extraction, plasmid concentration determination, and establishment of standard curve of fluorescence quantitative PCR, the corresponding kinetic curve was obtained, and the corresponding standard curve was calculated. During the clinical trial, DNA was extracted from chicken throat swab every day for detection.

### Establishment of CO_CL_

According to the distribution range of MIC, pathogenic MG strains were selected according to the following criteria: the highest peak MIC, the CO_WT_, the CO_PD_, MIC_50_, and MIC_90_ MIC strains. According to the specific distribution of MIC, five MIC strains were selected for clinical infection treatment.

At present, the relevant international organizations have not yet published a unified procedure for the establishment of CO_CL_. According to the reported methods, three analytical methods can be used to analyze clinical efficacy data, including “Window” algorithm, non-linear regression in SPSS software, and CART analysis in Alford Predictive Modeler software.

### Establishment of the CBP

After the CO_WT_, CO_PD_, and CO_CL_, the CBP was determined by making a tree chart according to the CBP. Firstly, the CO_WT_, CO_PD_, and CO_CL_ were compared. When the CO_WT_ was equal to the CO_CL_, the CO_WT_ was the CBP. When the situation was unequal, the three critical values were compared to determine the final CBP.

### Effect of Tylosin on the Lung Microbiota of MG Infected Chickens

The effect of tylosin on the lung microbiota of MG infected chickens was studied by 16S rRNA gene sequencing. In order to study the species composition diversity of the samples, the effective tags of all samples were clustered, and the sequences were clustered into OTUs with 97% consistency. The community structure and species abundance of each sample were plotted according to the classification. By analyzing the OTU (97% similarity) composition of different samples, the differences and distances of samples are reflected, and the differences of multidimensional data are reflected on the 2D/3D coordinate map. The coordinate axis takes the first two eigenvalues with the largest contribution to the square difference. If the distance between the two samples is close, the species composition of the two samples is similar.

## Results

### MIC Distribution of Tylosin Against MG and CO_WT_ Calculation

**FIGURE 1:**
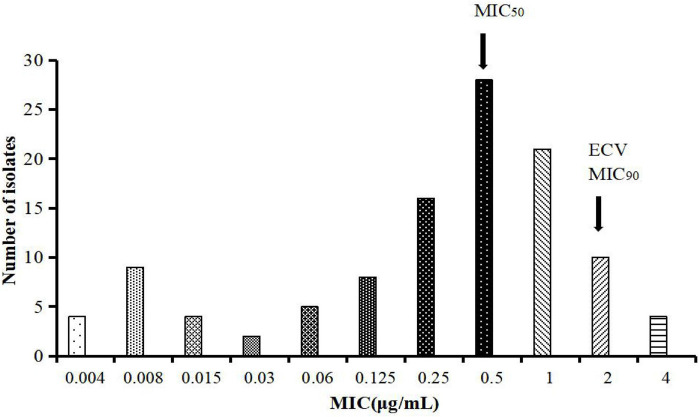
MICs distribution of tylosin against 111 strains of MG.

The MIC of tylosin against MG ranged from 0.004 to 4 μg/ml; the MIC_50_ and MIC_90_ were calculated as 0.5 and 2 μg/ml, respectively (Figure 1). The MIC distribution of tylosin against MG was substituted into raw count by ECOFFinder software. Four different endpoints (95, 97.5, 99, and 99.5%) were calculated as 2, 2, 4, and 4 μg/ml, respectively. Generally, the CO_WT_ should be set to contain at least 95% of wild-type strains, and the CO_WT_ of tylosin against MG was 2 μg/ml.

### Selection of Strains and *in vitro* PD Study

Ten MG strains of MIC_90_ were selected to evaluate the virulence by chicken embryo virulence test. In the chicken embryo virulence test, M17 showed a higher and faster lethality to chicken embryos. Because M17 had possessed the strongest virulence with the obvious infected symptoms, it was selected for the PD experiment.

The MIC of tylosin against M17 in FM-4 broth was 2 μg/ml, MBC was 4 μg/ml, and MPC was 5.12 μg/ml. The PAE of 1 h incubation was 1.55–2.32 h and that of 2 h incubation was 4.24–4.92 h.

**FIGURE 2:**
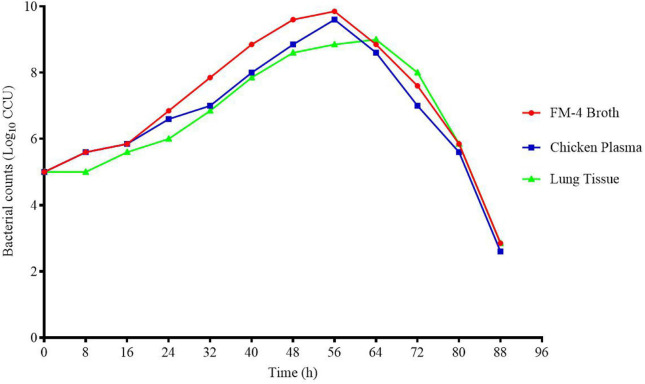
Growth curve of M17 in FM-4 broth (*in vitro*) plasma (*ex vivo*) and lung tissue (*ex vivo*).

The growth curves of M17 in FM-4 broth, plasma, and lung tissue *ex vivo* are shown in Figure 2. It can be seen from the figure that MG grows slowly, reaches the highest point at about 56 h, and then the content of viable bacteria decreases rapidly.

**FIGURE 3:**
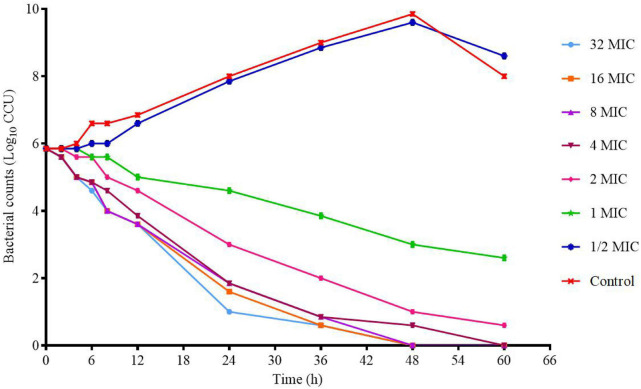
*In vitro* killing–time curves of tylosin against M17.

The killing–time curve of tylosin against M17 *in vitro* is shown in Figure 3. When the drug concentration is less than 4 × MIC, the curve shows concentration-dependent antibiotic activity. When the drug concentration is greater than 4 × MIC, the curve shows time-dependent antibiotic activity. The optimal bactericidal activity shows that the threshold is about 4 × MIC, and when the concentration is further increased, a similar effect is caused. This result is consistent with previous studies (Huang et al., 2018).

**FIGURE 4:**
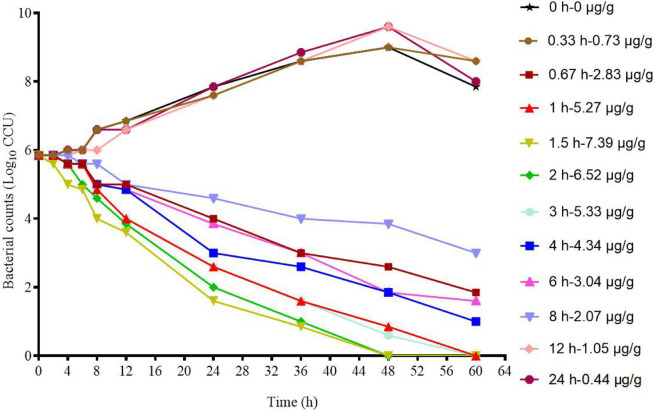
*Ex vivo* killing–time curves of tylosin against M17 in infected lung tissue.

The *ex vivo* killing–time curve of tylosin against MG in infected lung tissue is shown in Figure 4. Compared with the *in vitro* killing–time curve, the drug concentration was less than 4 × MIC, and it showed a concentration-dependent antibiotic activity.

### PK Study of Tylosin in Chickens

**FIGURE 5:**
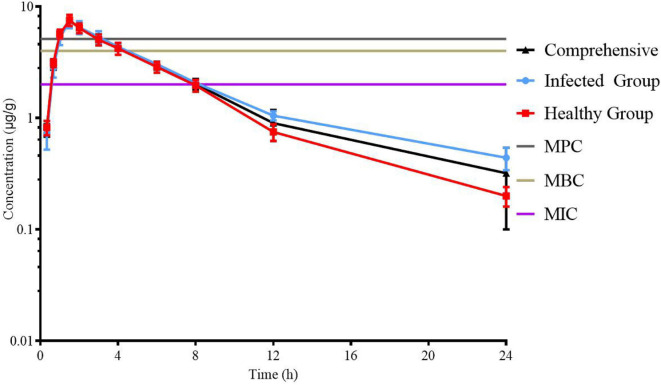
Semi-logarithmic time curve of tylosin (25 mg/kg b.w.) in lung tissue of chicken by gastric administration.

**FIGURE 6:**
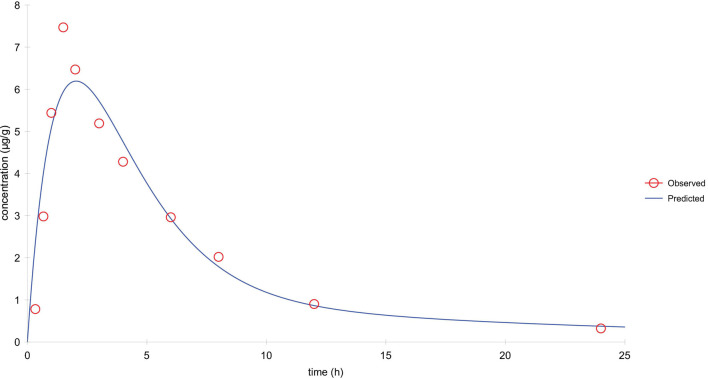
The drug–time curve of tylosin in lung tissue in comprehensive simulation.

Lung tissue samples were sampled at 0, 0.33, 0.67, 1, 1.5, 2, 3, 4, 6, 8, 12, 24, 36, and 48 h after a single oral administration of tylosin 25 mg/kg b.w. in healthy and infected chickens. The drug concentration after HPLC detection is shown in Table 1. It can be seen from Table 1 that there is no significant difference between the data of the healthy group and the data of the infected group and can be comprehensively analyzed. The data were brought into WinNonlin to establish the model of tylosin in chicken lung tissue, and the first-order absorption two-compartment model was used. The semi-logarithmic time curve of tylosin (25 mg/kg b.w.) in the lung tissue of chicken by gastric administration is obtained in Figure 5, the drug–time curve of tylosin in lung tissue in comprehensive simulation is shown in Figure 6, and the PK parameters are listed in Table 2.

**TABLE 1:** Drug concentration in lung tissues of chickens at different time points after intragastric administration of 25 mg/kg b.w. tylosin.

Time (h)	Concentration (μg/g)		
	Healthy group (*n* = 4)	Infected group (*n* = 4)	Comprehensive (*n* = 8)
0.33	0.82 ± 0.12	0.73 ± 0.11	0.78 ± 0.10
0.67	3.12 ± 0.25	2.83 ± 0.35	2.98 ± 0.27
1	5.68 ± 0.52	5.27 ± 0.47	5.44 ± 0.32
1.5	7.55 ± 0.87	7.39 ± 0.64	7.47 ± 0.24
2	6.42 ± 0.65	6.52 ± 0.52	6.47 ± 0.37
3	5.05 ± 0.57	5.33 ± 0.37	5.19 ± 0.38
4	4.21 ± 0.52	4.34 ± 0.35	4.28 ± 0.25
6	2.87 ± 0.32	3.04 ± 0.21	2.96 ± 0.24
8	1.96 ± 0.24	2.07 ± 0.12	2.02 ± 0.22
12	0.75 ± 0.13	1.05 ± 0.09	0.90 ± 0.28
24	0.20 ± 0.04	0.44 ± 0.03	0.32 ± 0.22
36	ND	ND	ND
48	ND	ND	ND

*“ND” means not detected.*

**TABLE 2:** Lung tissue pharmacokinetic parameters of healthy and infected chickens after intragastric administration of 25 mg/kg b.w. tylosin.

Parameter	Units	Healthy group	Infected group	Comprehensive
A	μg/g	6.92 ± 1.27	19.57 ± 2.36	9.07 ± 1.57
B	μg/g	0.05 ± 0.03	0.04 ± 0.03	0.03 ± 0.03
α	1/h	0.46 ± 0.22	0.50 ± 0.13	0.51 ± 0.17
β	1/h	0.41 ± 0.09	0.03 ± 0.02	0.05 ± 0.03
K01	1/h	0.51 ± 0.23	0.51 ± 0.33	0.53 ± 0.26
K10	1/h	0.46 ± 0.10	0.26 ± 0.12	0.31 ± 0.12
K12	1/h	0.001 ± 0.00	0.21 ± 0.11	0.17 ± 0.09
K21	1/h	0.41 ± 0.21	0.06 ± 0.05	0.08 ± 0.6
T_1/2K01_	h	1.37 ± 0.54	1.36 ± 0.63	1.32 ± 0.67
T_1/2__α_	h	1.51 ± 0.58	1.40 ± 0.66	1.36 ± 0.54
T_1/2__β_	h	1.70 ± 0.34	21.74 ± 0.47	13.86 ± 1.32
T_max_	h	2.08 ± 0.31	2.11 ± 0.27	2.05 ± 0.34
C_max_	μg/g	6.28 ± 0.42	6.17 ± 0.31	6.19 ± 0.63
AUC_24h_	hμg/g	35.57 ± 0.85	60.95 ± 0.97	51.13 ± 1.26
CL/F	L/h/kg	0.70 ± 0.36	0.41 ± 0.25	0.98 ± 0.23
Vd/F	L/kg	1.54 ± 0.58	1.58 ± 0.36	3.15 ± 0.46

*A and B, *y*-axis intercept terms; α and β, exponential coefficients; K01, absorption rate constant; K10, central compartment elimination rate constant; K12, distribution rate constant from central to peripheral compartment; K21, distribution rate constant from peripheral to central compartment; T_1/2K01_, absorption half-life of the drug; T_1/2__α_, half-life of α phase; T_1/2__β_, half-life of β phase; T_*max*_ is the peak time; C_*max*_ is the peak concentration; AUC_24h_ is the area under the curve of the drug; CL/F indicates the body clearance corrected for bioavailability; Vd/F, apparent volume of distribution based on the terminal elimination phase.*

### PK-PD Integration Modeling

The PK-PD parameters T > MIC, AUC_24h_/MIC, and C_max_/MIC of target organs were obtained by intragastric administration of tylosin in chickens at 25 mg/kg b.w. (Table 3). Because the bactericidal effect of tylosin in chickens was concentration dependent, the (AUC_24h_/MIC)ex parameter was selected to fit the PK-PD model *in vivo*.

**TABLE 3:** The PK-PD parameters of target organs in chickens.

PK-PD parameter	Company	Health Group	Infected group
C_max_/MIC	–	3.142	3.087
AUC_24h_/MIC	h	17.785	30.475
T > MIC	h	7.21	7.93
C_max_/MPC	–	1.227	1.206
AUC_24h_/MPC	h	6.947	11.904
T > MPC	h	2.62	2.71

The inhibitory Sigmoid E_max_ model was used to predict the relationship between PK-PD parameters and antimicrobial effects *in vivo*. Software fitting results of EC_50_ and N and target values under different effect targets are shown in Table 4.

**TABLE 4:** The results of *ex vivo* PK-PD modeling.

Parameter	Company	Health Group	Infected group
E_max_	Log_10_CCU	2.17	2.20
E_0_	Log_10_CCU	–4.59	–4.53
E_max_ – E_0_	Log_10_CCU	6.76	6.73
EC_50_	h	27.40	27.68
N	–	2.04	1.99
AIC	–	4.51	0.75
R^2^	–	0.9946	0.9957
(AUC_24h_/MIC)_ex_ for Bacteriostatic Action (E = 0)	h	18.98	19.25
(AUC_24h_/MIC)_ex_ for Bactericidal Action (E = −3)	h	48.84	51.19
(AUC_24h_/MIC)_ex_ for Eradication Action (E = −4)	h	86.59	95.26

### Establishment of the CO_PD_

**FIGURE 7:**
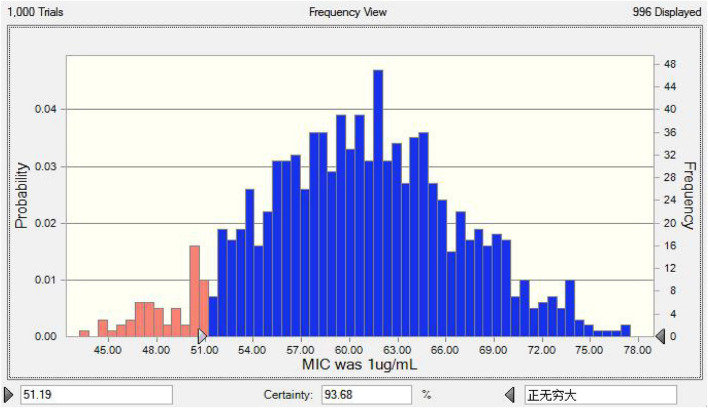
The predicted TAR of tylosin against MG at 1 μg/mL.

Using Crystalball 7 software, the AUC mean and standard deviation of lung tissue PK data were simulated by the Monte Carlo method, and the PK data of 10,000 chickens was obtained. The corresponding PK target value of E = –3 in the infected group was 51.19 by the WinNonlin simulation, and the results of reaching the standard under different MICs are shown in Figure 7. The simulation results of infected target sites were more meaningful to the establishment of the threshold; the final CO_PD_ of tylosin against MG was 1 μg/ml.

### Dose Regimen

The binding rate of tylosin in chicken lung tissue was generally about 9.47% by equilibrium dialysis method, and the free drug proportion was 91.5%. When the MIC value of the target strain was 2 μg/ml, the results of each dose calculated are shown in Table 5.

**TABLE 5:** Dosage for different drug purposes.

Antibacterial effect	b.w. (mg/kg)	Lifting dose (mg/kg)
Preventive dose	17.25	170
Therapeutic dose	45.88	460
Eradication dose	85.37	850

**FIGURE 8:**
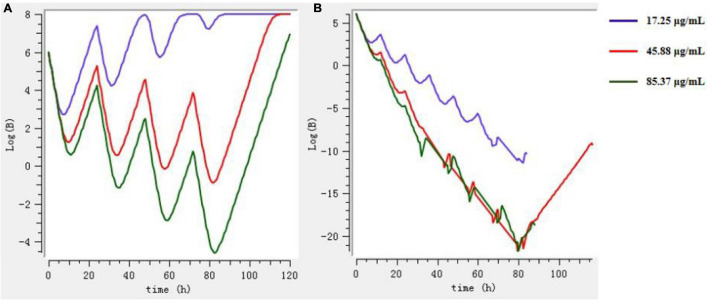
The growth of bacteria under different drug regimens by Mlxplore simulation. **(A)** 3 days at intervals of 24 h. **(B)** 3 days at intervals of 12 h.

The Mlxplore software was used to simulate and predict the growth of bacteria at three doses (prevention, treatment, and clearance) and at different intervals of administration, so as to obtain the best dosage regimen and interval of administration. According to Figure 8, the final dosage regimen is at 24-h intervals, 45.88 mg/kg b.w. dosage, and 3 days of continuous administration.

### Determination of DNA in Chickens Throat Swab Samples

To establish a fluorescent quantitative PCR for detection of MG, and to facilitate the detection of MG in throat swabs collected in clinical trials, the standard curve parameters E = 99.5, R^2^ = 0.990, Slope = −3.335, and y-int = 39.184 were obtained. The standard curve equation *y* = –3.335*x* + 39.184 was obtained. The detection limit for DNA copy numbers was 10^2^ kb.

**FIGURE 9:**
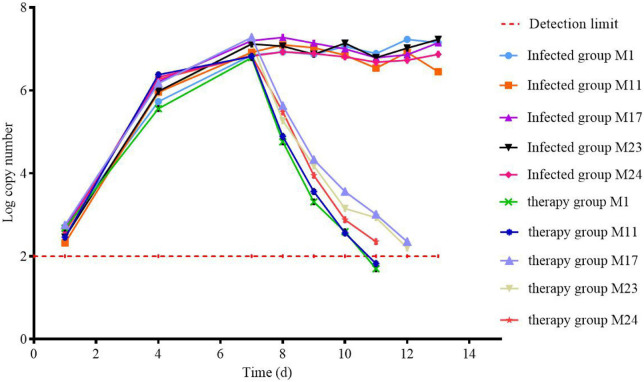
Changes in the amount of MG in chickens during clinical trials.

DNA was extracted from the throat swabs of chicken during all the experiments to calculate the copy number (Figure 9). It can be seen that MG was not detected in chickens before the experiment, indicating that there was no MG in chickens or the content was very low, which was below the detection limit. After the beginning of artificial infection, the content of MG in chickens began to increase, and reached the highest level on the seventh day of the last day of artificial infection. After treatment, the difference between the treatment group and the infection group was obvious. The content of MG in the treatment group decreased rapidly after administration. On the fourth day, there was the existence of MG, which was not detected in the group. The content of MG in the infected group reached a relatively stable state and fluctuated at a certain level.

### Establishment and Verify the CO_CL_

The results of clinical animal experiments are shown in Table 6. After drug treatment, the cure rate of sensitive strains M1 and M11 can reach 100%. The cure rate of strains with relatively large MIC was lower than that of sensitive strains. The higher the MIC value, the lower the cure rate. This shows that the therapeutic effect of drugs is negatively correlated with the MIC value of infected strains. When the MIC of the infected strain was 0.03 μg/ml, the cure rate was 100%. When the MIC of the infected strain was 0.5 μg/ml, the cure rate was 93.3%. When the MIC of the infected strain was 1 μg/ml, the cure rate was 80%. According to the CO_CL_, maximum MIC value should be chosen when the cure rate is greater than or equal to 90%, so the CO_CL_ of tylosin against MG was 0.5 μg/ml.

**TABLE 6:** Therapeutic effect of tylosin on different MIC MG.

Group	Strain label	MIC (μg/ml)	Number of animals	Average score	Reduction rate (%)	Effective rate (%)	Cure rate (%)
Blank group	–	–	15	0.20	–	–	–
Infection group	M1	0.008	15	6.67	–	–	–
	M11	0.03	15	7.27	–	–	–
	M24	0.5	15	7.6	–	–	–
	M23	1	15	7.53	–	–	–
	M17	2	15	7.73	–	–	–
Treatment group	M1	0.008	15	0.73	89.1	100	100
	M11	0.03	15	1.2	83.5	100	100
	M24	0.5	15	1.73	77.2	100	93.3
	M23	1	15	1.93	74.4	87.7	80.0
	M17	2	15	2	74.1	80	73.3

The data obtained was analyzed by the “Windo W” method, and the parameters of MaxDiff and CAR were determined. The CO_CL_ range was determined according to the parameters. The probabilities obtained by CAR calculation method in this study are 1, 1, 0.97, 0.92, and 0.84, respectively. Finally, the MIC value of the gradient smaller than the maximum MIC was chosen as the upper limit of critical value, which was 1 μg/ml. The corresponding values calculated by MaxDiff algorithm are 11, 13, 11, 3, and 0, respectively. Then, the MIC distribution corresponding to the maximum MaxDiff value, chosen as the lower limit of the CO_CL_, was 0.03 μg/ml. The distribution range of CO_CL_ obtained by this method was 0.03–1 μg/ml. The CO_CL_ of clinical cure test was 0.5 μg/ml was in this range.

Then, according to the formula between POC and MIC proposed by EUCAST, data obtained are fitted by non-linear regression in SPSS software. The model expression was *y* = 82.992−9.364*x*−1.628*x*^2^−0.91*x*^3^, and the simulated R^2^ value was 0.975. According to the result expression, when the MIC is 0.57 μg/ml, it corresponds to 90% cure rate, so the CO_CL_ should be less than 0.57 μg/ml. The CO_CL_ of the clinical cure test was 0.5 μg/ml and was in this range.

Finally, data from clinical trials were brought into Salford Predictive Modeler software for CART analysis. Regression tree showed that the cure rate was 97.8% when MIC ≤ 0.75 μg/ml, 76.7% when MIC > 0.75 μg/ml, and 89.3% when MIC was close to 0.75 μg/ml. Therefore, the CO_CL_ obtained by CART analysis should be the closest and less than 0.75 μg/ml. The CO_CL_ of the clinical cure test was 0.5 μg/ml and was in this range.

### Establishment of the CBP

According to the decision tree based on CLSI, the CO_WT_ of tylosin against MG was 2 μg/ml, CO_PD_ was 1 μg/ml, and CO_CL_ was 0.5 μg/ml. The CBP of tylosin against MG is not the same, within three gradients. Taking these three critical values into the flow chart of the CBP published by CLSI conforms to CO_WT_ > CO_PD_ > CO_CL_. The corresponding CBP should choose the CO_WT_, which means the CBP of tylosin against MG was 2 μg/ml.

### The Effect of Tylosin on Lung Microbiota

**FIGURE 10:**
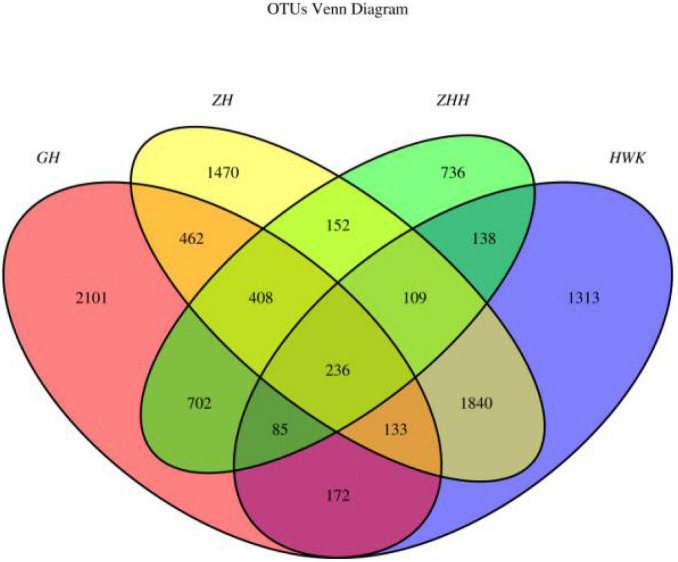
Venn diagrams of the core and unique OTUs of the four groups. HWK (control group); GH (infection group); ZH (treatment group); ZHH (post-treatment group).

The qualitative comparisons of the core and unique operational taxonomic units (OTUs) among the four groups are based on 97% sequence similarity and the Venn diagrams are shown in Figure 10. Representative sequences for each OTU were retrieved and classified to genus level. There were 4,026, 4,299, 4,810, and 2,566 OTUs identified in the HWK (control group), GH (infection group), ZH (treatment group), and ZHH (post-treatment group) group, respectively. A big change in the respiratory tract flora was observed in chickens after MG infection. The number of respiratory tract flora reached the highest after treatment and decreased significantly after cure. However, when we focused on the unique flora of each group, we found that the respiratory tract flora was the highest in the infection group. With the use of tylosin and after treatment, the number of unique flora in the respiratory tract has decreased significantly. The results showed that after the infection of MG, the bacteria colonized in the respiratory tract increased because of the decrease of immunity. When tylosin is used to treat respiratory tract infection, some of the bacteria whose value is fixed due to the decrease of immunity are rapidly reduced, while some of the original bacteria that are often in existence are greatly increased due to the availability of living space. However, when tylosin was used to cure MG infection, all kinds of bacteria groups were reduced to the minimum due to the use of drugs and enhancement of chicken immunity.

**FIGURE 11:**
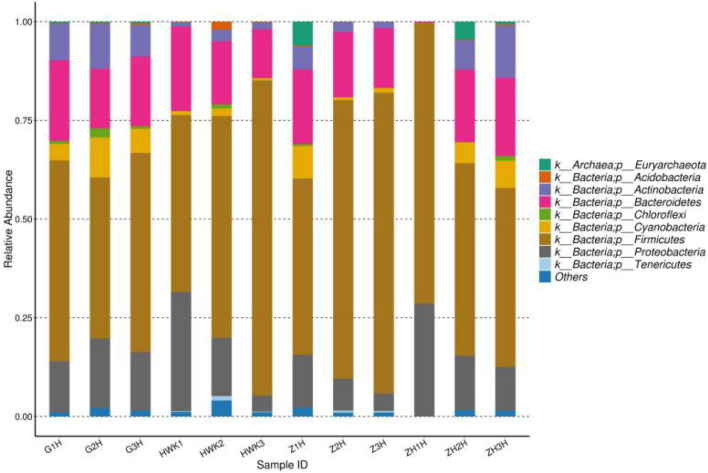
Taxonomic classification of the 16S rRNA sequences at phylum levels. HWK (control group); GH (infection group); ZH (treatment group); ZHH (post-treatment group).

The change of lung microbiota in the phylum level is shown in Figure 11. In total, 37 phyla were identified in the four groups, of which 14 phyla had an abundance of more than 1%. *Firmicutes* was the most dominant phylum (44.78–79.82% abundance). *Proteobacteria* and *Bacteroidetes* constituted 4.05–30.19% and 12.37–21.40%, respectively. In the whole clinical trial process, *Firmicutes* showed a change of recovery after decreasing first and then increasing, *Bacteroidetes* showed little change, and *Proteobacteria* showed an increasing trend after decreasing. MG is a Gram-negative bacterium, but it does belong to *Firmicutes*. During the period of MG infection, the amount of *Firmicutes* decreased because of the competition of *Mycoplasma*. During the treatment period, *Mycoplasma* decreased significantly, and other *Firmicutes* recovered. After cure, it tends to return to the initial state. *Bacteroidetes* is a Gram-negative bacterium, and tylosin is less effective against this bacterium, so *Bacteroides* has little change during the whole test period. *Proteobacteria* include *Escherichia coli* and *Salmonella*, and tylosin has a significant antibacterial effect on these bacteria. Therefore, the number of *Proteobacteria* may be reduced after being artificially infected with MG and treated with tylosin until it recovers after cure.

**FIGURE 12:**
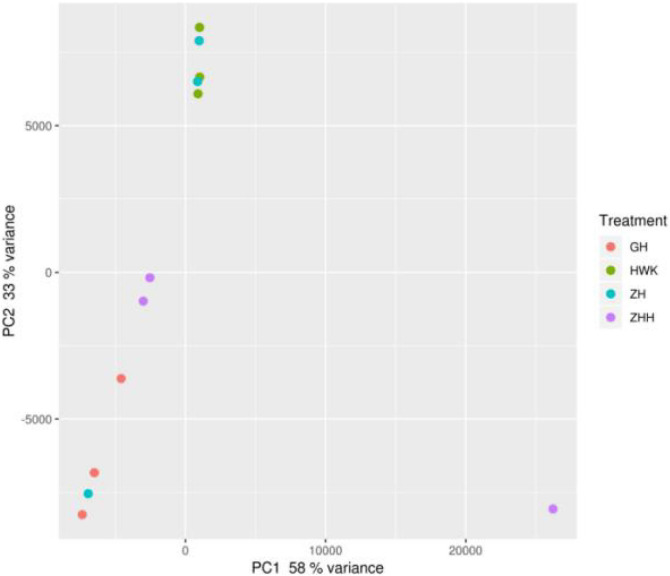
PCA of the lung microbiome of chickens. HWK (control group); GH (infection group); ZH (treatment group); ZHH (post-treatment group).

In addition, beta diversity was also estimated to further understand the variations of the lung microbiota community. The principal coordinate analysis (PCoA) plots were obtained and shown in Figure 12. When the abnormal samples were removed, there were obvious differences among each group, and there were similarities within the groups.

## Discussion

*Mycoplasma gallisepticum* is a highly pathogenic *mycoplasma* of poultry, which has great harm to chicken breeding industry (Wijesurendra et al., 2015; Xie et al., 2016). Standard detection methods are essential for the determination of drug sensitivity. The growth characteristics of bacteria determine the choice of detection methods, because MG has a long growth cycle and small colony formation, which is not suitable for paper dilution method and agar dilution method, so the main test method is micro broth dilution method (Kohner et al., 1997).

In terms of bacterial resistance, there are differences in time and place, and the results of sensitivity test are also related to detection methods and strain sources (Garnacho-Montero et al., 2005). EUCAST and CLSI believe that the development of CO_WT_ requires at least 100 isolates per bacterial species, but it has also been reported that only at least 30 wild-type bacteria are needed to develop CO_WT_ (Smith and Kronvall, 2014; Toutain et al., 2019). In order to ensure that the selected strains are sufficiently representative, 111 strains of MG were isolated from several provinces in China in 1 year; thus, there are a large number of isolated strains that came from a wide range of sources. The MIC value of tylosin against 111 strains of MG ranged from 0.004 to 4 μg/ml. The result was similar to that of Jong et al., who determined the MICs of tylosin against 82 MG strains, and the MICs ranged from 0.004 to 8 μg/ml (Jong et al., 2020).

The wild-type isolates should not have any acquired anti-mutation mechanism by establishing the CO_WT_ (Peng et al., 2016). From the columnar distribution of MIC, it can be seen that the MIC of tylosin against MG is mostly in a normal distribution; in addition, there are a few sensitive strains. Conservatively, the strains used in this laboratory are all wild-type strains, and all the MIC results can be used. The data used to establish the CO_WT_ include the diameter of bacteriostatic zone (mm) and MIC (μg/ml), but most of them are based on MIC data to establish the critical value of wild type. If the critical value is determined by the diameter of the bacteriostatic zone, the conversion relationship between the diameter of bacteriostatic zone and MIC needs to be given (Turnidge and Paterson, 2007). In this study, the broth microdilution method was used for sensitivity test, which could be directly used to determine the CO_WT_. CO_WT_ was formulated as 2 μg/ml. The result was similar to that of Landman et al. (2008) who derived the unofficial CBP of S ≤ 1 and R ≥ 4 μg/ml for tylosin against MG (Landman et al., 2008; Kreizinger et al., 2017; El-Hamid et al., 2019).

In previous reports, the concentration of drugs in infected sites was highly correlated with blood concentration (Craig, 1998), but in later studies, the PK-PD model was used to optimize the detection of drug concentration in infected sites (Lei et al., 2017). Because tylosin has good intracellular activity and can accumulate in cells (Mattoes and Nightingale, 2002), it has good therapeutic effect on intracellular bacteria such as MG (Rauf et al., 2014), so the concentration of drug in lung tissue is used in PK-PD research. Moreover, the concentration of tylosin in lung tissue is much higher than that in plasma, which prevents drug resistance due to the concentration remaining in the drug resistance selection window for a long time (Nan et al., 2018). Comparing the results of the healthy group and the infected group, it showed that tylosin could stay longer in the infected group and play a better bactericidal role. This result is consistent with the result of tilmicosin in healthy and infected chickens (Zhang et al., 2020). The infected group was chosen to construct the PK-PD model since it is more in line with the actual treatment situation.

The bactericidal properties of antibiotics are closely related to the combination of biological drugs. The same drug may have different bactericidal properties for different bacterial strains. Most macrolides exhibit time-dependent antimicrobial activity, and the parameters of T > MIC and AUC/MIC are usually selected for the PK-PD model (Owens and Ambrose, 2007). In this study, the experimental method of the PK-PD model construction is to draw a bactericidal curve in half-body conditions, and the logarithmic value of bacterial quantity change is used as a PD index. The bactericidal curve showed that the type of antimicrobial activity of tylosin against MG was time-dependent. Therefore, AUC_24h_/MIC was selected as the parameter of PK-PD *in vivo*. This result is different from the concentration-dependent antibacterial activity of tilmicosin (Huang et al., 2019).

The PK-PD integrated model can be used to optimize the reasonable dosage scheme of veterinary doctors (Toutain et al., 2002). This study optimized the dosage regimen of tylosin for the treatment of MG infection to be 45.88 mg/kg b.w. once a day for three consecutive days. In order to achieve the best clinical efficacy, the daily dose of tylosin should not be high or low.

When formulating the CO_CL_, the influence on the results mainly comes from the MIC and POC. With good clinical data, clinical cutoff values can be quickly established. However, when the clinical data are complex, it is necessary to rely on the analysis of various software to help establish the CO_CL_. The CO_CL_ obtained by different methods in this study were all 5 μg/ml or within this range. Judging from the results, the results obtained by different algorithms were similar, indicating that different methods have the same goal under the condition of good experimental data. However, under the conditions of different results, the CO_CL_ that meets all the conditions should be selected (Xu et al., 2021).

The formulation of the CO_WT_ is mainly based on the MIC of bacteria to drugs *in vitro*; the formulation of the CO_PD_ is mainly based on the determination of AUC at the target site and the elimination of drug distribution and metabolism in animals; the formulation of the CO_CL_ is subject to the sample size of animals, whether animals are completely healthy or not, and the administration of drugs. The influence of many factors, such as quantity, will have different results in clinical practice. Because of the different emphasis of these three methods, the values obtained are different. The final CBP of tylosin against MG was 2 μg/ml in this study, which is two concentration gradients different from the CBP 8 μg/ml of erythromycin to *Ureaplasma urealyticum* published by CLSI (Clinical And Laboratory Standards Institude, 2011), indicating that the resistance of tylosin against MG is better than that of erythromycin against *U. urealyticum*.

The critical value of this study is CO_WT_ > CO_PD_ > CO_Cl_, which indicates that there may be some drug-resistant MG in clinic, and tylosin may have no effect on some clinical MG when used in clinic. VetCAST recommends CO_WT_ as a surrogate when the CBP is not established (Toutain et al., 2017). This result is consistent with previous reports, while CO_WT_ > CO_PD_, CO_WT_ also plays an important role in establishing the CBP (Lei et al., 2018; Mi et al., 2021). Tylosin, as a commonly used drug in clinical treatment of MG infection, has shown a very good effect on MG infection. Therefore, it is very important to protect the existing effectiveness of tylosin.

The 16S rDNA sequencing method has been used to study a lot of bacteria in the digestive tract of chickens (Stanley et al., 2014; Waite and Taylor, 2014), but few in the lung microbiota. In mammals, the composition of respiratory microbiota is associated with disease severity (Piters et al., 2016) and the risk of developing respiratory diseases in the future (Teo et al., 2015), and it is believed that this may also be the case in avian animals. Sequence analysis showed that there were many kinds of bacteria in chicken lung microbiota. According to the phylum analysis, *Firmicutes* is the dominant flora of lung microbiota, followed by *Proteobacteria* and *Bacteroidetes*, which is the same as that of Ying Zhou (Zhou et al., 2020). From the OTUs results, the results showed that the number of lung microbiota increased after infection. The lung microbiota reached the highest after treatment and decreased significantly after cure. However, when we focused on the unique flora of each group, we found that the lung microbiota was the highest in the infected group. With the use and treatment of tylosin, the number of unique lung microbiota decreased significantly. In addition, in the analysis of PCoA, it can be seen that the gap between the groups is very obvious. In general, the lung microbiota of chickens infected with MG and treated with tylosin changed significantly. The results showed that tylosin not only was effective against MG, but also could kill many kinds of bacteria in the lung microbiota of chickens. In addition, other studies have also reported the fact that antibiotic treatment leads to a decrease in taxa. Therefore, in the project of using antibiotics to treat bacterial infection, we should consider not only the effect of antibiotics but also the impact on the microbial community in the organism.

## Conclusion

It has been proven that the abuse of antibiotics will increase the resistance of antibiotics. In order to make rational use of antibiotics, it is necessary to establish a CBP and a reasonable administration plan. In this experiment, the CO_WT_ of tylosin against MG was 2 μg/ml by establishing a drug resistance test method to detect whether MG was resistant to tylosin; the CO_PD_ of tylosin against MG was 1 μg/ml by PD and PK; through clinical trials of different MICs of MG, the CO_CL_ of tylosin against MG was 0.5 μg/ml, and the flow chart was established according to the CBP value given by CLSI to determine the drug resistance standard of tylosin against MG, which was 2 μg/ml. Finally, a reasonable drug delivery plan was given by the PK-PD model with a 24-h interval and 45.88 mg/kg b.w. was administered continuously for 3 days. This study also proved that drug treatment has a great influence on lung microbiota, and we should also take this influence into account when we choose therapeutic drugs.

## Data Availability Statement

The data presented in the study are deposited in the National Center for Biotechnology Information (NCBI) repository, accession number is PRJNA745254.

## Ethics Statement

The animal study was reviewed and approved by Institutional Animal Care and Use Committee at HZAU. Written informed consent was obtained from the owners for the participation of their animals in this study.

## Author Contributions

AH, SW, ZY, and HH conceived and designed the experiments. AH, SW, JG, and YG performed the experiments. AH, SW, JG, YG, and JL analyzed the data. LH, XW, YT, ZL, ZY, and HH contributed reagents or materials or analysis tools. AH and HH wrote the manuscript. All authors contributed to the article and approved the submitted version.

## Conflict of Interest

The authors declare that the research was conducted in the absence of any commercial or financial relationships that could be construed as a potential conflict of interest.

## Publisher’s Note

All claims expressed in this article are solely those of the authors and do not necessarily represent those of their affiliated organizations, or those of the publisher, the editors and the reviewers. Any product that may be evaluated in this article, or claim that may be made by its manufacturer, is not guaranteed or endorsed by the publisher.
